# Supplementary material for: Effects of different exercise types and cycles on pain and quality of life in breast cancer patients: A systematic review and network meta-analysis
Source: PLoS One. 2024 Jul 3;19(7):e0300176. doi: 10.1371/journal.pone.0300176 (PMC11221662; doi:10.1371/journal.pone.0300176)
Supplement: Attachment — Submitted filename: PONE-D-23-35809_Comments.docx [file pone.0300176.s003.docx]

PONE-D-23-35809

Reviewer Comments

The manuscript is well-written that only needs minor revision in the following;

Introduction: The manuscript contains an elaborate literature review, but definitions of the key concepts should be included. Some operational definitions are included in discussion section, I think it is better to move to introduction.

How about the operational definition of breast cancer? Shall we describe in introduction section?

Is there any categorization or staging of breast cancer? Any articles describe whether there is the difference in QoL or dependent variables according to stage of the breast cancer? Were the studies reviewed on all types or stages of breast cancer? Shall we include some information about these?

There may be inclusion criteria for certain types of exercises assigned to breast cancer patients in these reviewed RCTs. The authors may need to elaborate more on this. E.g., Which stage of breast cancer patients are assigned to YOGA, and so on. I am curious which stages of breast cancers included for exercise therapy.

“Breast cancer's prevalence has steadily risen over recent decades, notably among younger age groups.” – please add citation, and could you please describe what age groups 15-24 or something age group?

Methodology – perfectly done. Sincerely I have limited experiences with review and network meta-analysis, so that I don’t have special comments on this section.

Conclusion section is not clear and concise. It still describes some data and CI. I recommend to revise this section to better and clear understanding by the readers.

Thanks to authors for the good manuscript.

Sorry for late submission.
