# Supplementary material for: Effects of different exercise types and cycles on pain and quality of life in breast cancer patients: A systematic review and network meta-analysis
Source: PLoS One. 2024 Jul 3;19(7):e0300176. doi: 10.1371/journal.pone.0300176 (PMC11221662; doi:10.1371/journal.pone.0300176)
Supplement: Attachment — Submitted filename: Response to Reviewers.docx [file pone.0300176.s004.docx]

Shanxi University

92 Wucheng Road

Taiyuan, Shanxi, China

Email: wondergod1027@163.com

Dec 22, 2023

Dear reviewers:

Thank you for your comments concerning our manuscript. Those comments are all valuable and very helpful for revising and improving our paper, as well as the important guiding significance to our researches. We have studied comments carefully and have made correction which we hope meet with approval. Revised portion are marked in red in the paper. The main corrections in the paper and the responds to the reviewer’s comments are as flowing:

**Reviewer #1:** There are many errors in write up e.g. the referrence is missing

**Response:** Thank you very much for these constructive comments and we have double-checked and revised.

- Training for 12 weeks falls under which category?

**Response:** This 12-week training is short-term and is described at the end of the first paragraph of the "2.4 Data Extraction and Definitions" section.

- Referrence for Jadad scale –

**Response:** Thank you for your suggestion. We have added references for the Jadad scale.

KIM S Y, KIM K N, KIM D W, etc. Reporting Quality Analysis of Randomized Controlled Trials in Journal of Neurosurgical Anesthesiology: A Methodological Assessment[J/OL]. Journal of Neurosurgical Anesthesiology, 2021, 33(2): 154. https://doi.org/10.1097/ANA.0000000000000662.

Table 2- please write particioants (n)- because if not, not sure what partcipants mean

- Arrangement- Table 2 after Figure 1?- not tally with text

**Response:** Thank you for your suggestion. we have made changes to address the above issues in line with your suggestions.

- Discussion may need to be improved. Mostly expalin the results of the review, may suggest to include abou what do you think cause the certain exercise is better than others- with supported literature

**Response:** Thank you for your comment. We have optimised and modified the discussion section.

**Reviewer #2:** The manuscript is well-written that few minor revisions needed. The methodology looks strong and perfect. Please revise accordingly in the attached file. Thanks for the interesting manuscript. I think the conclusion should be clear and concise, and give recommendations for effective utilization of your study findings.

1.Introduction: The manuscript contains an elaborate literature review, but definitions of the key concepts should be included. Some operational definitions are included in discussion section, I think it is better to move to introduction.

**Response:** Thank you for your comments. We have adjusted and revised the conclusion and discussion sections.

2.How about the operational definition of breast cancer? Shall we describe in introduction section?

**Response:** Thank you for your comments. The study population was operationally defined as patients with stage I-III breast cancer, aged ≥18 years, who had been diagnosed and completed surgery. We have added it to the introductory section.

3. Is there any categorization or staging of breast cancer? Any articles describe whether there is the difference in QoL or dependent variables according to stage of the breast cancer? Were the studies reviewed on all types or stages of breast cancer? Shall we include some information about these?

**Response:** Breast cancer patients with stage I-III were included in our study, but it was not possible to count the amount of effect in breast cancer patients with different stages. During our review, we did not find any relevant literature reporting significant differences in quality of life among breast cancer patients with different stages, and we will continue to monitor this issue. The studies we reviewed involved breast cancers of I-III, and some of the descriptions have been modified in the Inclusion Exclusion Criteria section.

4. There may be inclusion criteria for certain types of exercises assigned to breast cancer patients in these reviewed RCTs. The authors may need to elaborate more on this. E.g., Which stage of breast cancer patients are assigned to YOGA, and so on. I am curious which stages of breast cancers included for exercise therapy.

**Response:** None of the literature we included had detailed stages or analyses of breast cancer patients, so we were unable to analyse and elaborate in more depth.

5. “Breast cancer's prevalence has steadily risen over recent decades, notably among younger age groups.” – please add citation, and could you please describe what age groups 15-24 or something age group?

**Response:** Thank you for your comment. We found from data from two Global Cancer Surveys that the minimum average age of breast cancer patients decreased from 31.3 years in 2018 to 29.7 years in 2020, but we did not find more detailed statistics by age group.

BRAY F, FERLAY J, SOERJOMATARAM I, etc. Global cancer statistics 2018: GLOBOCAN estimates of incidence and mortality worldwide for 36 cancers in 185 countries[J/OL]. CA: A Cancer Journal for Clinicians, 2018, 68(6): 394-424. https://doi.org/10.3322/caac.21492.

SUNG H, FERLAY J, SIEGEL R L, etc. Global Cancer Statistics 2020: GLOBOCAN Estimates of Incidence and Mortality Worldwide for 36 Cancers in 185 Countries[J/OL]. CA: A Cancer Journal for Clinicians, 2021, 71(3): 209-249. https://doi.org/10.3322/caac.21660.

6. Conclusion section is not clear and concise. It still describes some data and CI. I recommend to revise this section to better and clear understanding by the readers.

**Response:** Thank you for your suggestion. we have made changes to address the above issues in line with your suggestions.

**Reviewer #3:** Introduction, 1st paragraph:

1."Additionally, a systematic review... [9] [10]." If there is a systematic review about QoL in Breast cancer patients, why have you performed another one? Please refer the interest or innovation of yours here, which I understand that exists.

**Response:** Thank you for your comment. In previous studies researchers only analysed the types of exercise and did not compare different exercises. In our study it was necessary to determine the effects of different exercise cycles and different types of exercise on patients' quality of life and other aspects of health.

2.Methods, Study selection

The exclusion criteria (b) also considered excluding conference papers published with peer-review? Please be more specific.

3. Methods, Data extraction and definition (last parapgraph)

"If the required data could not... excluded from consideration." - I hope that the exclusion was just for meta-analysis and that it was considered in the systematic review. Please be more specific.

4. Methods, Data synthesis and analysis (second paragraph)

Consider changing to "A p-value greater or equal to 0.05 indicated that ….. Conversely, if the p-value was less or equal to 0.05…"

5. Results

1st paragraph: "eventually, a total of 36 papers… (Error! Reference source not found.)… screening process is shown in (Error! Reference source not found.)"

6. All the tables - normalize the number of significant digits (consider using integers just for counts, 2 decimal places for means and standard deviations, 3 decimal digits for p-values) and align text on the left, number on the right

7. All the manuscript - use the p-value in small caps, an always consider p > 0.05 instead of p > 0.05 in all the sentences where it appears

**Response:** Thank you very much for these constructive comments and we have made changes to address the above issues in line with your suggestions.

We hope that these changes will meet with your approval. If you have any other questions, please do not hesitate to send us your comments.

Sincerely,

Jin Dong

De-sheng Wang
