# Supplementary material for: Effects of different exercise types and cycles on pain and quality of life in breast cancer patients: A systematic review and network meta-analysis
Source: PLoS One. 2024 Jul 3;19(7):e0300176. doi: 10.1371/journal.pone.0300176 (PMC11221662; doi:10.1371/journal.pone.0300176)
Supplement: Attachment — Submitted filename: Response to Reviewers.docx [file pone.0300176.s005.docx]

Shanxi University

92 Wucheng Road

Taiyuan, Shanxi, China

Email: wondergod1027@163.com

Jan 27, 2024

Dear reviewers:

Thank you for your comments concerning our manuscript. Those comments are all valuable and very helpful for revising and improving our paper, as well as the important guiding significance to our researches. We have studied comments carefully and have made correction which we hope meet with approval. Revised portion are marked in red in the paper. The main corrections in the paper and the responds to the reviewer’s comments are as flowing:

**Reviewer #1:**

**Q1:** In the abstract, what do you mean by “anti-rent” exercise?

**Response:** Sorry, this is a spelling error, we mean "resistance exercise". Thank you for checking, we have corrected it.

**Q2:** In the introduction, suggest to describe about what does it mean by usual care.

**Response:** Thanks for your suggestion, we have included an explanation of usual care in the introduction.

**Q3:** Basically the exercise modalities compared were only aerobic, resistance and yoga. Any consideration to include other mode of exercise?

**Response:** Yes, the only types of exercise we are currently comparing are aerobic exercise, resistance exercise and yoga. Because these three exercises are currently more studied, and are easier to implement in clinical practice. In future studies, we will consider adding more exercise methods for comparison to improve the breadth of the study.

**Q4:** Fatigue was not mention in the beginning as one of the outcome that the author wants to study.

**Response:** Thank you for asking, but we do mention the fatigue as a secondary outcome measure in the paragraph on “**2.3 outcome”**.

**Q5:** How does the short and long duration exercise program been decided? Any literature to support this?

**Response:** Yes, there are literature on long and short term exercise. Here are two references:

a) <https://doi.org/10.1016/j.jshs.2022.12.008>.
b) https://doi.org/[10.1038/nrneurol.2017.128](https://doi.org/10.1038/nrneurol.2017.128).

**Q6:** Selection criteria for the article: patients with breast cancer, did you include patients pre and post-surgery?

**Response:** In **2.2 Study selection**, we defined breast cancer patients as "patients with stage 1-3 breast cancer over 18 years old who had **undergone** surgery" in the inclusion and exclusion criteria.

**Q7：**Suggest to specify, resistance exercise- type of exercise, does it involves the upper limb exercise or non-specific. How about muscle endurance exercise, does it included in the analysis?

**Response:** Thanks for your suggestion, we have included an explanation of different types of exercise, including resistance exercise, in "2.4Data extraction and definition". For muscular endurance exercise, we classified it as resistance exercise for analysis.

Sincerely,

De-sheng Wang

Jin Dong
