# Supplementary material for: Effects of different exercise types and cycles on pain and quality of life in breast cancer patients: A systematic review and network meta-analysis
Source: PLoS One. 2024 Jul 3;19(7):e0300176. doi: 10.1371/journal.pone.0300176 (PMC11221662; doi:10.1371/journal.pone.0300176)
Supplement: Attachment — Submitted filename: Response to Reviewers.docx [file pone.0300176.s006.docx]

Shanxi University

92 Wucheng Road

Taiyuan, Shanxi, China

Email: wondergod1027@163.com

Jan 31, 2024

Dear reviewers:

Thank you for your comments concerning our manuscript. Those comments are all valuable and very helpful for revising and improving our paper, as well as the important guiding significance to our researches. We have studied comments carefully and have made correction which we hope meet with approval. Revised portion are marked in red in the paper. The main corrections in the paper and the responds to the reviewer’s comments are as flowing:

**Reviewer #1:**

**Q1:** In the abstract, what do you mean by “anti-rent” exercise?

**Response:** Sorry, this is a spelling error, we mean "resistance exercise". Thank you for checking, we have corrected it.

**Q2:** In the introduction, suggest to describe about what does it mean by usual care.

**Response:** Thanks for your suggestion, we have included an explanation of usual care in the introduction.

**Q3:** Basically the exercise modalities compared were only aerobic, resistance and yoga. Any consideration to include other mode of exercise?

**Response:** Yes, the only types of exercise we are currently comparing are aerobic exercise, resistance exercise and yoga. Because these three exercises are currently more studied, and are easier to implement in clinical practice. In future studies, we will consider adding more exercise methods for comparison to improve the breadth of the study.

**Q4:** Fatigue was not mention in the beginning as one of the outcome that the author wants to study.

**Response:** Thank you for asking, but we do mention the fatigue as a secondary outcome measure in the paragraph on “**2.3 outcome”**.

**Q5:** How does the short and long duration exercise program been decided? Any literature to support this?

**Response:** Yes, there are literature on long and short term exercise. Here are two references:

a) <https://doi.org/10.1016/j.jshs.2022.12.008>.
b) https://doi.org/[10.1038/nrneurol.2017.128](https://doi.org/10.1038/nrneurol.2017.128).

**Q6:** Selection criteria for the article: patients with breast cancer, did you include patients pre and post-surgery?

**Response:** In **2.2 Study selection**, we defined breast cancer patients as "patients with stage 1-3 breast cancer over 18 years old who had **undergone** surgery" in the inclusion and exclusion criteria.

**Q7：**Suggest to specify, resistance exercise- type of exercise, does it involves the upper limb exercise or non-specific. How about muscle endurance exercise, does it included in the analysis?

**Response:** Thanks for your suggestion, we have included an explanation of different types of exercise, including resistance exercise, in "2.4Data extraction and definition". For muscular endurance exercise, we classified it as resistance exercise for analysis.

**Reviewer #2:** The manuscript is well-written that few minor revisions needed. The methodology looks strong and perfect. Please revise accordingly in the attached file. Thanks for the interesting manuscript. I think the conclusion should be clear and concise, and give recommendations for effective utilization of your study findings.

**Q1:**Introduction: The manuscript contains an elaborate literature review, but definitions of the key concepts should be included. Some operational definitions are included in discussion section, I think it is better to move to introduction.

**Response:** Thank you for your comments. We have adjusted and revised the conclusion and discussion sections.

**Q2:**How about the operational definition of breast cancer? Shall we describe in introduction section?

**Response:** Thank you for your comments. The study population was operationally defined as patients with stage I-III breast cancer, aged ≥18 years, who had been diagnosed and completed surgery. We have added it to the introductory section.

**Q3:** Is there any categorization or staging of breast cancer? Any articles describe whether there is the difference in QoL or dependent variables according to stage of the breast cancer? Were the studies reviewed on all types or stages of breast cancer? Shall we include some information about these?

**Response:** Breast cancer patients with stage I-III were included in our study, but it was not possible to count the amount of effect in breast cancer patients with different stages. During our review, we did not find any relevant literature reporting significant differences in quality of life among breast cancer patients with different stages, and we will continue to monitor this issue. The studies we reviewed involved breast cancers of I-III, and some of the descriptions have been modified in the Inclusion Exclusion Criteria section.

**Q4:** There may be inclusion criteria for certain types of exercises assigned to breast cancer patients in these reviewed RCTs. The authors may need to elaborate more on this. E.g., Which stage of breast cancer patients are assigned to YOGA, and so on. I am curious which stages of breast cancers included for exercise therapy.

**Response:** None of the literature we included had detailed stages or analyses of breast cancer patients, so we were unable to analyse and elaborate in more depth.

**Q5:** “Breast cancer's prevalence has steadily risen over recent decades, notably among younger age groups.” – please add citation, and could you please describe what age groups 15-24 or something age group?

**Response:** Thank you for your comment. We found from data from two Global Cancer Surveys that the minimum average age of breast cancer patients decreased from 31.3 years in 2018 to 29.7 years in 2020, but we did not find more detailed statistics by age group.

BRAY F, FERLAY J, SOERJOMATARAM I, etc. Global cancer statistics 2018: GLOBOCAN estimates of incidence and mortality worldwide for 36 cancers in 185 countries[J/OL]. CA: A Cancer Journal for Clinicians, 2018, 68(6): 394-424. https://doi.org/10.3322/caac.21492.

SUNG H, FERLAY J, SIEGEL R L, etc. Global Cancer Statistics 2020: GLOBOCAN Estimates of Incidence and Mortality Worldwide for 36 Cancers in 185 Countries[J/OL]. CA: A Cancer Journal for Clinicians, 2021, 71(3): 209-249. https://doi.org/10.3322/caac.21660.

**Q6:** Conclusion section is not clear and concise. It still describes some data and CI. I recommend to revise this section to better and clear understanding by the readers.

**Response:** Thank you for your suggestion. we have made changes to address the above issues in line with your suggestions.

Sincerely,

De-sheng Wang

Jin Dong
