# Supplementary material for: Effects of different exercise types and cycles on pain and quality of life in breast cancer patients: A systematic review and network meta-analysis
Source: PLoS One. 2024 Jul 3;19(7):e0300176. doi: 10.1371/journal.pone.0300176 (PMC11221662; doi:10.1371/journal.pone.0300176)
Supplement: Attachment — Submitted filename: PONE-D-23-35809_R3 Comments.docx [file pone.0300176.s008.docx]

PONE-D-23-35809 R3

Reviewer Comments

The manuscript is thoroughly revised by the authors addressing reviewers’ comments, but formatting and typo errors should be fine-tuned.

Firstly, thanks for the authors’ effort for better manuscript.

**Introduction**:

1. “Breast cancer's prevalence has steadily risen over recent decades, notably among younger age groups.” This sentence still makes confusing. I think, according to authors’ response, it should be “The prevalence of breast cancer has steadily risen over recent decades (from ….. to ….. during 20.. to 20.. if you have exact information). The age of diagnosing breast cancer becomes younger ………… probably diagnostic facilities and access to healthcare are improving in recent years or if you have relevant information – please add.
2. Inclusions by the reviewed RCTs - Completed surgery for how long? Any categorizations for duration of completed surgery? Durations of illness they suffering may have effects on their QoL, for chronic diseases, the longer the disease duration the poorer QoL in general. Reverse or how about your findings on QoL in these reviewed RCTs? Could you please explain here if you have reviewed and found these facts? Or if not, you may add as weakness or bias or recommend to explore more information in further studies.
3. Shall we describe shortly on standard care for control groups (CG) that you have described in comparison in the later sections?

Thanks to authors for the better manuscript.
