# Supplementary material for: Effects of different exercise types and cycles on pain and quality of life in breast cancer patients: A systematic review and network meta-analysis
Source: PLoS One. 2024 Jul 3;19(7):e0300176. doi: 10.1371/journal.pone.0300176 (PMC11221662; doi:10.1371/journal.pone.0300176)
Supplement: Attachment — Submitted filename: Response to Reviewers.docx [file pone.0300176.s009.docx]

Shanxi University

92 Wucheng Road

Taiyuan, Shanxi, China

Email: wondergod1027@163.com

Feb 20, 2024

Dear reviewers:

Thank you for your comments concerning our manuscript. Those comments are all valuable and very helpful for revising and improving our paper, as well as the important guiding significance to our researches. We have studied comments carefully and have made correction which we hope meet with approval. Revised portion are marked in red in the paper. The main corrections in the paper and the responds to the reviewer’s comments are as flowing:

**R1:**

**Q1:** “Breast cancer's prevalence has steadily risen over recent decades, notably among younger age groups.” This sentence still makes confusing. I think, according to authors’ response, it should be “The prevalence of breast cancer has steadily risen over recent decades (from ….. to ….. during 20.. to 20.. if you have exact information). The age of diagnosing breast cancer becomes younger ………… probably diagnostic facilities and access to healthcare are improving in recent years or if you have relevant information – please add.

**Response:** Thank you for your suggestion. I have reviewed the literature again and revised the part in the introduction. “The age of diagnosing breast cancer becomes younger …… probably diagnostic facilities and access to healthcare are improving in recent years. "We have adjusted these sentences in previous revisions, Mainly through the information obtained in Reference 2.

**Q2:** Inclusions by the reviewed RCTs - Completed surgery for how long? Any categorizations for duration of completed surgery? Durations of illness they suffering may have effects on their QoL, for chronic diseases, the longer the disease duration the poorer QoL in general. Reverse or how about your findings on QoL in these reviewed RCTs? Could you please explain here if you have reviewed and found these facts? Or if not, you may add as weakness or bias or recommend to explore more information in further studies.

**Response:** Your suggestion is very good, however, only a small number of literatures in our included literature mentioned how long to complete surgery for exercise, so we could not perform a categorical analysis of time to complete surgery. As for the relationship between duration of illness and quality of life in chronic patients, there are also insufficient data for analysis in our study, and we will supplement these weaknesses in limitation.

**Q3:** Shall we describe shortly on standard care for CG that you have described in comparison in the later sections?

**Response:** Sure. We defined control measures as usual care, including medication as necessary, health education, general stretching exercises, and passive movements with the help of a physician. We have added this information to the introduction in previous revisions.

Sincerely,

De-sheng Wang

Jin Dong
